# p53 expression in patients with ulcerative colitis - associated with dysplasia and carcinoma: a systematic meta-analysis

**DOI:** 10.1186/s12876-017-0665-y

**Published:** 2017-10-25

**Authors:** Xiaohong Lu, Yuanjie Yu, Shiyun Tan

**Affiliations:** Departmemt of gastroenterology, Renmin Hospital of Wuhan University, Jiefang Road 238, Wuhan, 430060 China

**Keywords:** p53, Ulcerative colitis, Dysplasia, Carcinoma

## Abstract

**Background:**

Tumor suppressor gene p53 expression has been reported in patients with ulcerative colitis (UC). However, the correlation between p53 expression and UC remains controversial. The aim of this meta-analysis was to investigate the association between p53 expression and different pathological types of UC.

**Methods:**

Publications were searched in the PubMed, Embase, EBSCO, Wangfang, and CNKI databases. The overall odds ratios (ORs) and their corresponding 95% confidence intervals (95% CIs) were summarized in this study.

**Results:**

Final 19 papers were identified in this meta-analysis, including 1068 patients with UC and 130 normal tissue samples. Immunohistochemical p53 expression was significantly higher in UC without dysplasia and carcinoma (UC group) compared to normal tissue samples (OR = 3.14, *P* = 0.001), higher in UC with dysplasia than in UC group (OR = 10.76, *P* < 0.001), and higher in UC with colorectal cancer (CRC) than in UC with dysplasia (OR = 1.69, *P* = 0.035). Subgroup analysis of ethnicity (UC group vs. normal tissues) showed that p53 expression was correlated with UC in Asians, but not in Caucasians. When UC with dysplasia was compared to UC group, p53 expression was linked to UC with dysplasia among both Asians and Caucasians. When UC-CRC was compared to UC with dysplasia, p53 expression was not associated with UC-CRC in both Caucasians and Asians.

**Conclusions:**

p53 expression was closely associated with UC-CRC development. p53 expression showed different ethnic characteristics among different pathological types of UC.

**Electronic supplementary material:**

The online version of this article (10.1186/s12876-017-0665-y) contains supplementary material, which is available to authorized users.

## Background

As one common pathological type of inflammatory bowel diseases (IBD), ulcerative colitis (UC) is characterized by a relapsing, idiopathic, and chronic inflammatory disease which usually affects the entire colon and rectum [1, 2]. The prevalence of this disease has been rising in the world, patients with UC are correlated with an increased risk of developing colorectal cancer (CRC) [3, 4].

Although the exact mechanism of UC is not fully understood, increasing evidence reports that genetic and environmental factors are important in the pathogenesis of UC [5–7]. Given the increased risk of CRC in UC, dysplasia is precancerous lesions, it is essential to clinical screening of UC patients with dysplasia [8]. The *p53*, a key tumor-suppressor gene (TSG), is mapped to the short arm of chromosome 17 (17p13) [9]. *p53* gene encodes the p53 protein and is responsible for the regulation of cell cycle, DNA repair, and apoptosis [10, 11]. The *p53* mutation is the most common event in human carcinomas, due to the accumulation of mutant *p53*, p53 protein often shows nuclear staining, which makes it easily detected using immunohistochemistry (IHC) method [12, 13]. The expression of p53 protein can be measured by IHC in different histological types of UC [14, 15].

Although Du et al. reported the association between KRAS and TP53 mutations with IBD-associated colorectal cancer [16], the correlation between p53 expression and the risk of dysplasia and cancer in patients with UC remained to be elucidated. Because the sample size of an individual study was small in UC, moreover, there were some inconsistent results with respect to p53 expression in UC. For example, Li 2004 et al. reported that no immunoreactivity for p53 expression was found in UC and normal tissue samples [17]. Klump 1997 et al. reported that p53 expression was noted in UC without dysplasia/carcinoma and UC with dysplasia [15]. Thus, we first conducted this meta-analysis involving more eligible articles to determine the correlation between p53 expression and different histological types of UC, including normal tissue samples, UC without dysplasia/carcinoma, UC with dysplasia, and UC with CRC.

## Methods

### Search strategy

A systematic literature search was conducted in the PubMed, Embase, EBSCO, Wangfang, and CNKI databases prior to February 13th, 2017. We used the relevant key words and search terms to identify eligible papers: (inflammatory bowel disease OR ulcerative colitis) AND expression AND (TP53 OR p53 OR p53 protein). The references of the included papers were also carefully checked to get other potential studies.

### Study selection

The eligible publications should meet the following inclusion criteria: 1) patients were diagnosed with UC by pathological examination; 2) normal tissue samples belonged to normal control group, UC groups included UC without dysplasia/carcinoma, UC with dysplasia, and UC with colorectal cancer; 3) studies on the immunohistochemical determination of p53 protein expression provided sufficient information to determine the relationship between p53 expression and UC. Only the most complete article with more information was selected in this meta-analysis when authors published multiple articles using the same study population.

### Data extraction and quality assessment

According to the above inclusion criteria, two independent authors reviewed and extracted information from the eligible articles: first author’s surname, year of publication, country, number of patients, cut-off values, rate of p53 expression, staining location, and the total number of patients in UC and normal control groups. Any controversial issue was discussed by all authors. The quality of the eligible publications was assessed based on the Newcastle-Ottawa Scale (NOS), ranging from 0 to 9. Studies with 6 or more scores were considered to be of high quality [18].

### Statistical analysis

Stata software, version 12.0 (STATA Corp., College Station, TX, USA) was used in this meta-analysis. The overall odds ratios (ORs) and their corresponding 95% confidence intervals (95% CIs) were calculated to estimate the strength of the relationship between p53 expression and UC risk. Possible heterogeneity among reported studies was measured using the Cochran’s Q statistic [19]. The random-effects model was chosen in the present study. A *P* value of <0.1 for the Q-test showed a substantial heterogeneity, a sensitivity analysis was conducted to evaluate the influence of one study on the pooled results by omitting a single study [20]. For the pooled data with more than eight studies, Egger’s test was performed to determine possible publication bias [21].

## Results

### Characteristics of the eligible studies

**Fig. 1:**
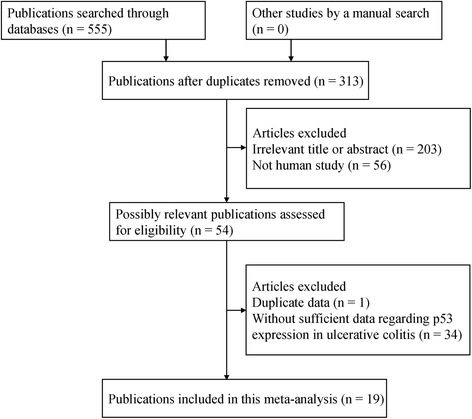
Flowchart summarizing literature search strategy and selection of studies

As indicated in Fig. 1, 555 potential papers were initially searched from a range of online electronic databases (PubMed, Embase, EBSCO, Wangfang, and CNKI). According to the above study selection, final 19 articles published from 1993 to 2013 [14, 15, 17, 22–37] were identified in the current meta-analysis, including 1068 patients with UC and 130 normal tissue samples. Nine studies evaluated the correlation of p53 expression in UC versus normal tissue samples. 11 studies evaluated the correlation of p53 expression in UC with dysplasia versus UC without dysplasia and carcinoma. 16 studies evaluated the relationship of p53 expression in UC with carcinoma versus UC with dysplasia. All studies met a score of equal to or greater than 6 in this meta-analysis (Additional file 1: Table S1). Table 1 lists the baseline characteristics of the included studies.

**Table 1:** General characteristics of the eligible publications in this study

First author	Country	Ethnicity	Age	Cut-off (IHC)	Location	Normal	UC	UC with dysplasia	UC with cancer	NOS
						N (E%)	N (E%)	N (E%)	N (E%)	
Taylor 1993 [29]	UK	Caucasians	NA	NA	Nucleus		20 (0)	20 (30)	21 (52.4)	9
Harpaz 1994 [28]	USA	Caucasians	NA	10%	Nucleus			40 (62.5)	56 (60.7)	7
Klump 1997 [15]	Germany	Caucasians	NA	0%	Nucleus		68 (2.9)	27 (63)	5 (100)	8
Fogt 1998 [27]	USA	Caucasians	NA	0%	Nucleus			10 (90)	8 (87.5)	7
Sato 1999 [26]	Japan	Asians	NA	NA	Nucleus	35 (14.3)	105 (41)	55 (67.3)	11 (90.9)	6
Hirota 2000 [25]	Japan	Asians	NA	Focal or diffuse	Nucleus			40 (60)	13 (92.3)	6
Ishitsuka 2001 [24]	Japan	Asians	NA	Local or diffuse	Nucleus		23 (0)	13 (61.5)	4 (75)	6
Brüwer 2002 [23]	Germany	Caucasians	NA	0%	Nucleus	10 (0)	15 (6.7)	16 (37.5)	14 (57.1)	9
Li 2004 [17]	China	Asians	44	10%	Nucleus	25 (0)	5 (0)	14 (21.4)	2 (50)	7
Yoshida 2004 [22]	Japan	Asians	NA	Scattered	Nucleus	7 (28.6)	19 (26.3)	46 (63)	7 (57.1)	7
Wang 2005 [30]	China	Asians	44	1%	Nucleus		25 (4)	7 (42.9)	8 (50)	8
Wang 2008 [31]	China	Asians	32	5%	Nucleus	10 (10)	20 (15)			6
Alkim 2009 [37]	Turkey	Caucasians	46	0%	Nucleus	10 (40)	26 (88.5)			7
Kawamata 2011 [14]	Japan	Asians	28–60	NA	Nucleus	12 (0)	8 (12.5)	8 (62.5)	8 (75)	8
Tanaka 2011 [36]	Japan	Asians	49.5	Focal	Nucleus		28 (0)	81 (59.3)	10 (90)	6
Gushima 2011 [35]	Japan	Asians	49	10%	Nucleus			11 (72.7)	14 (21.4)	8
Scarpa 2013 [34]	Italy	Caucasians	51	NA	Nucleus	11 (9.1)	19 (5.3)	10 (20)	7 (57.1)	8
Shigaki 2013 [32]	Japan	Asians	26–80	5%	Nucleus			58 (46.6)	27 (59.3)	7
Wohl 2013 [33]	Czech Republic	Caucasians	NA	33%	Nucleus	10 (0)	16 (6.3)			7

*NA* not applicable, *UC* ulcerative colitis, *IHC* immunohistochemistry, *N* study population, *NOS* Newcastle-Ottawa Scale, *E* expression

### Association of p53 expression in UC without dysplasia and carcinoma versus normal tissue samples

**Fig. 2:**
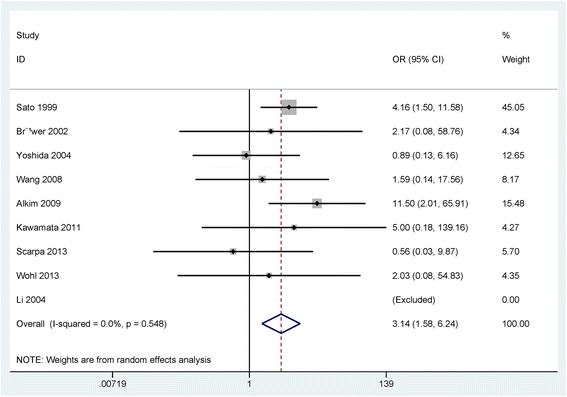
Forest plot of the pooled OR of p53 expression in UC without dysplasia or carcinoma versus normal tissue samples

The result of 233 patients with UC vs 130 normal tissue samples demonstrated that the level of p53 expression was notably increased in UC compared with normal tissue samples (OR = 3.14, 95% CI = 1.58–6.24, *P* = 0.001) (Fig. 2).

Subgroup analysis by ethnicity (Asians and Caucasians) showed that p53 expression was linked to Asians with UC (OR = 2.85, 95% CI = 1.25–6.47, *P* = 0.012), but was not correlated with Caucasians with UC (OR = 3.49, 95% CI = 0.85–14.34, *P* = 0.083) (Table 2).

**Table 2:** Subgroup analysis of ethnicity regarding p53 expression

Subgroup (ethnicity)	OR (95% CI)	Heterogeneity: *P*	*P* value
UC with cancer vs. UC with dysplasia			
Caucasian	1.60 (0.89–2.86)	0.459	0.116
Asians	1.65 (0.77–3.56)	0.104	0.198
UC with dysplasia vs. UC			
Caucasian	18.47 (5.62–60.71)	0.325	<0.001
Asians	8.28 (3.08–22.25)	0.071	<0.001
UC vs. normal tissues			
Caucasian	3.49 (0.85–14.34)	0.317	0.083
Asians	2.85 (1.25–6.47)	0.521	0.012

*UC* ulcerative colitis, *OR* odds ratio, *95% CI* 95% confidence interval

### Association of p53 expression in UC with dysplasia versus UC without dysplasia/carcinoma

**Fig. 3:**
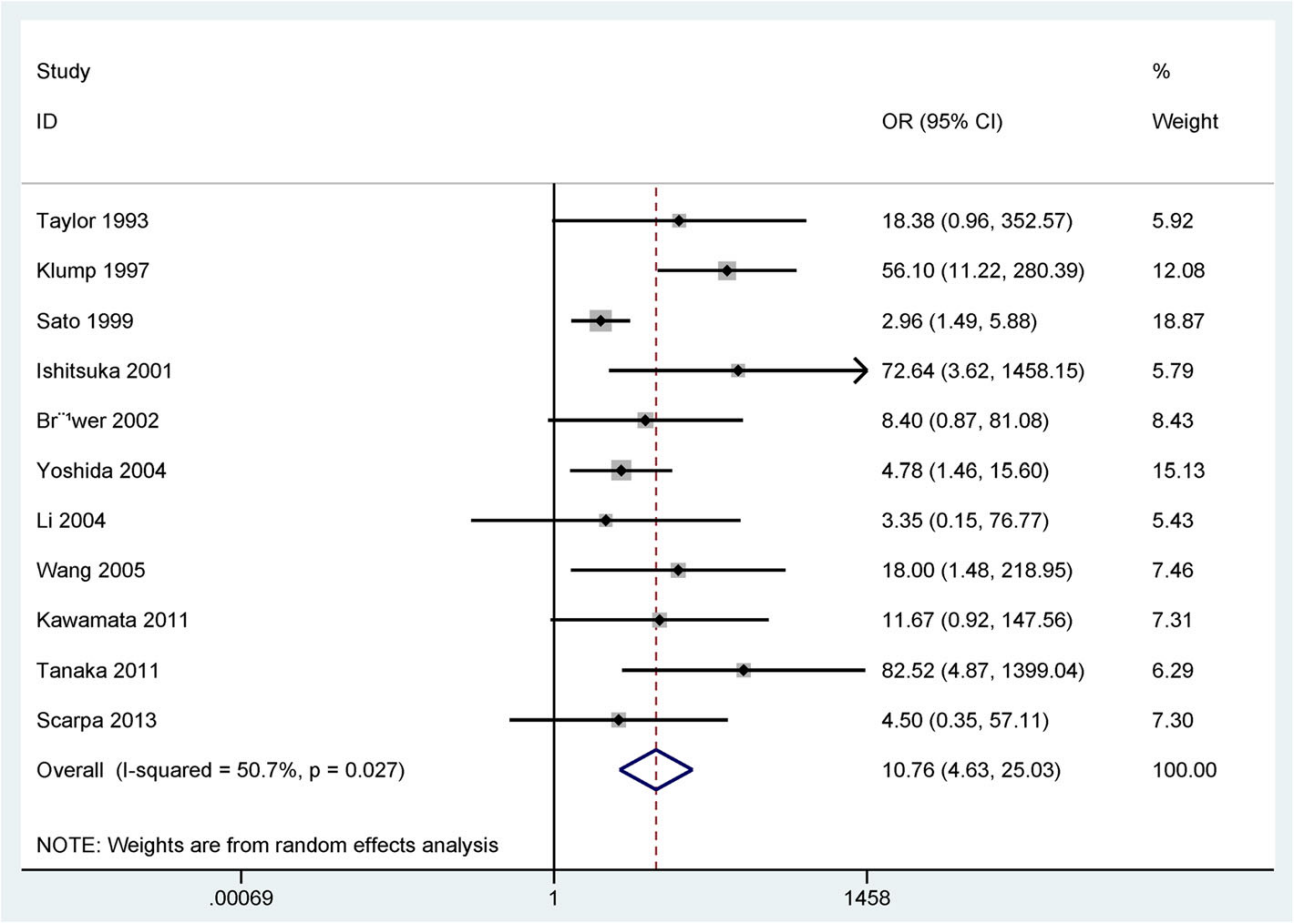
Forest plot of the pooled OR of p53 expression in UC with dysplasia versus without dysplasia or carcinoma

The result showed that the frequency of p53 expression was notably higher in UC with dysplasia than in UC without dysplasia/carcinoma (OR = 10.76, 95% CI = 4.63–25.03, *P* < 0.001), including 297 UC patients with dysplasia and 335 UC patients without dysplasia and carcinoma (Fig. 3).

According to ethnic population (Asians and Caucasians) (Table 2), subgroup analysis showed that p53 expression was correlated with UC with dysplasia in the Asian and Caucasian populations (OR = 8.28, 95% CI = 3.08–22.25, *P* < 0.001; OR = 18.47, 95% CI = 5.62–60.71, *P* < 0.001).

### Association of p53 expression in UC with carcinoma versus UC with dysplasia

**Fig. 4:**
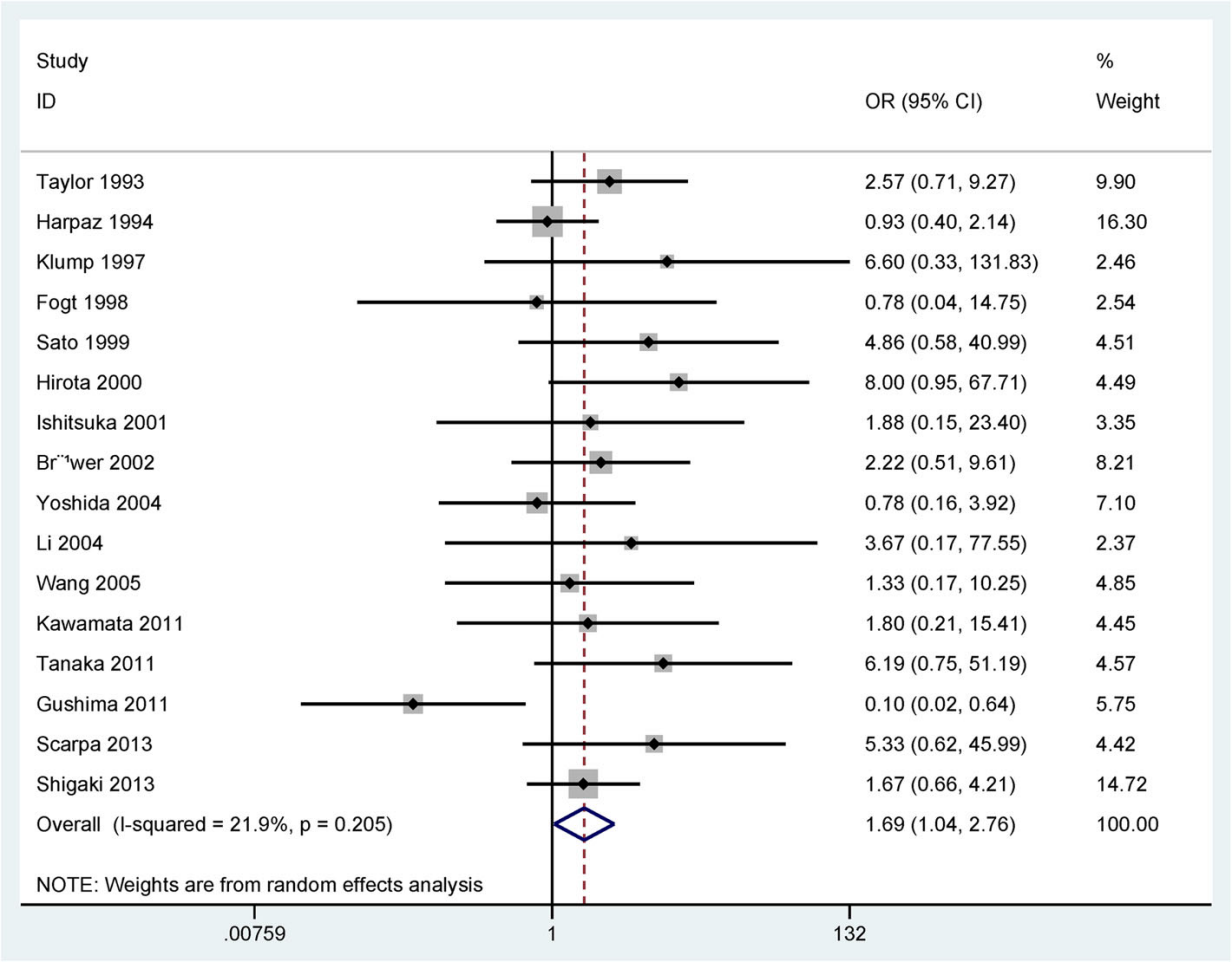
Forest plot of the pooled OR of p53 expression in UC with carcinoma versus with dysplasia

The result from the comparison of 215 UC with carcinoma and 456 UC with dysplasia indicated that the frequency of p53 expression in UC with carcinoma was significantly higher than in UC with dysplasia (OR = 1.69, 95% CI = 1.04–2.76, *P* = 0.035) (Fig. 4).

Subgroup analysis based on ethnicity (Asians and Caucasians) showed that p53 expression was not associated with UC-CRC in both Caucasians and Asians (OR = 1.60, 95% CI = 0.89–2.86, *P* = 0.116; OR = 1.65, 95% CI = 0.77–3.56, *P* = 0.198; respectively) (Table 2).

### Sensitivity analysis in UC with dysplasia versus UC without dysplasia/carcinoma

A slight heterogeneity was found in UC with dysplasia versus UC without dysplasia/carcinoma (*P* = 0.027). When one study (Klump 1997 et al. [15]) was removed, and the overall OR value was re-calculated (OR = 7.28, 95% CI = 3.48–15.21), *P* < 0.001), with no evidence of heterogeneity (*P* = 0.181).

### Publication bias

**Fig. 5:**
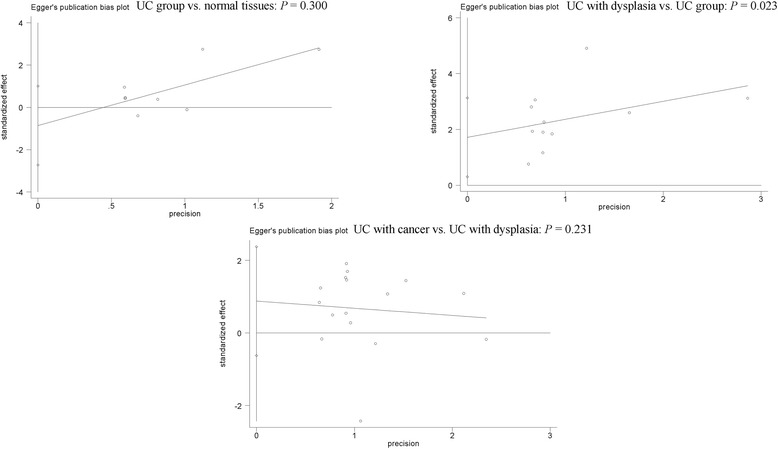
Forest plot of publication bias using Egger’s test

Egger’s test showed that no publication bias was observed in the comparison of UC without dysplasia/carcinoma and normal tissue samples and the comparison of UC with carcinoma and UC with dysplasia (*P* > 0.1) (Fig. 5). A slight publication bias was found in UC with dysplasia versus UC without dysplasia and carcinoma (*P* = 0.023 < 0.05) (Fig. 5).

## Discussion

TSG p53 expression has been frequently reported in human malignancies [38, 39]. Some studies suggest that positive expression of the p53 gene is linked to the development and progression of tumors [40–42]. Immunohistochemical analysis of p53 protein expression is also found in patients with UC [32, 34]. However, the results regarding p53 expression in different pathological types of UC are still inconsistent and conflicting. The present study was carried out to analyze whether p53 expression was associated with the increased risk of UC with dysplasia and carcinoma.

*p53* gene showed different expression levels in UC-associated different pathological types, ranging from 0% [14, 17, 23, 33] to 40% [37] in normal tissue samples, and ranging from 0% [17, 24, 29, 36] to 88.5% [37] in UC without dysplasia and carcinoma. The expression of the p53 gene had different frequencies in UC with dysplasia or carcinoma, with a range from 20% [34] to 90% [27] in UC with dysplasia, and with a range from 21.4% [35] to 100% [15] in UC with CRC. This study integrated all available studies involving a large population showed that immunohistochemical detection of p53 protein expression was significantly higher in UC with CRC than in UC with dysplasia, higher in UC with dysplasia than in UC without dysplasia and carcinoma, and higher in UC without dysplasia and carcinoma than in normal tissue samples, which suggested that p53 expression was closely linked to UC-CRC carcinogenesis and the progression of UC with dysplasia. In addition, a slight heterogeneity was detected in UC with dysplasia versus UC without dysplasia and carcinoma (*P* = 0.027 < 0.1). Thus, we conducted a sensitivity analysis to assess the change of the pooled OR and heterogeneity based on the omission of one study. We removed one study [15], and a re-calculated OR was not significantly changed, with no evidence of heterogeneity (*P* = 0.181), suggesting the stability of the current result. The reasons of heterogeneity from this study and other studies were not clear, perhaps due to the use of inappropriate or different conditions in IHC method, which may cause an observed bias.

Subgroup analysis of ethnicity (the Asian and Caucasian populations) was performed to find different correlation among different subgroups. When UC without dysplasia and carcinoma was compared to normal tissue samples, the result of subgroup analysis revealed that only Asians were susceptible to p53 expression. When UC with dysplasia was compared to UC without dysplasia and carcinoma, the result showed that the Asian and Caucasian populations were susceptible to p53 expression. When UC with carcinoma was compared to UC with dysplasia, the result demonstrated that Asians and Caucasians were not susceptible to p53 expression.

Some limitations should be noted in the present meta-analysis. First, although a range of online electronic databases was systematically searched to identify available publications. Egger’s test demonstrated a slight publication bias in UC with dysplasia vs. UC without dysplasia and carcinoma. Only papers published in English or Chinese were included in this meta-analysis. The unpublished articles or conference abstracts were excluded because of insufficient information. Articles with positive conclusions were usually published than these articles with negative conclusions. Second, the main ethnic groups consisted of Asians and Caucasians in this study, but other study populations, such as African population, were lacking. Finally, three eligible studies reported the data between p53 expression and dysplasia and cancer in patients with Crohn’s disease (CD) [37, 43, 44], more studies with large sample sizes are essential to further perform a meta-analysis to assess the correlation of p53 expression in CD in the future.

## Conclusions

This study suggests that p53 gene has a notably higher expression frequency in UC-CRC than in UC with dysplasia, higher expression level in UC with dysplasia than in UC without dysplasia and carcinoma, and higher expression rate in UC without dysplasia and carcinoma than in normal tissue samples. Further large-scale clinical trials with large sample sizes are needed to validate our conclusions in the future.

## Additional file

Additional file 1: Table S1. Study quality using Newcastle-Ottawa-Scale (NOS). (DOCX 17 kb)

## Abbreviations

Cis: Confidence intervals
CRC: Colorectal cancer
IBD: Inflammatory bowel diseases
IHC: Immunohistochemistry
NOS: Newcastle-Ottawa Scale
ORs: Odds ratios
TSG: Tumor-suppressor gene
UC: Ulcerative colitis
