# Supplementary material for: p53 expression in patients with ulcerative colitis - associated with dysplasia and carcinoma: a systematic meta-analysis
Source: BMC Gastroenterol. 2017 Oct 25;17:111. doi: 10.1186/s12876-017-0665-y (PMC5655860; doi:10.1186/s12876-017-0665-y)
Supplement: Additional file 1: Table S1. — Study quality using Newcastle-Ottawa-Scale (NOS). (DOCX 17 kb) [file 12876_2017_665_MOESM1_ESM.docx]

**Table S1. Study quality using Newcastle-Ottawa-Scale (NOS).**

| Study | Selection | Comparability | Exposure/Outcome | Quality score |
| --- | --- | --- | --- | --- |
| Taylor 1993 | ★★★★ | ★★ | ★★★ | 9 |
| Harpaz 1994 | ★★★★ | ★★ | ★ | 7 |
| Klump 1997 | ★★★★ | ★ | ★★★ | 8 |
| Fogt 1998 | ★★★★ | ★★ | ★ | 7 |
| Sato 1999 | ★★★★ | ★ | ★ | 6 |
| Hirota 2000 | ★★★ | ★ | ★★ | 6 |
| Ishitsuka 2001 | ★★★ | ★ | ★★ | 6 |
| Brüwer 2002 | ★★★★ | ★★ | ★★★ | 9 |
| Li 2004 | ★★★★ | ★ | ★★ | 7 |
| Yoshida 2004 | ★★★★ | ★ | ★★ | 7 |
| Wang 2005 | ★★★★ | ★ | ★★★ | 8 |
| Wang 2008 | ★★★ | ★ | ★★ | 6 |
| Alkim 2009 | ★★★★ | ★ | ★★ | 7 |
| Kawamata 2011 | ★★★★ | ★★ | ★★ | 8 |
| Tanaka 2011 | ★★★ | ★ | ★★ | 6 |
| Gushima 2011 | ★★★★ | ★★ | ★★ | 8 |
| Scarpa 2013 | ★★★★ | ★ | ★★★ | 8 |
| Shigaki 2013 | ★★★★ |  | ★★★ | 7 |
| Wohl 2013 | ★★★★ | ★ | ★★ | 7 |
